# West Nile Virus Lineage 2 in Horses and Other Animals with Neurologic Disease, South Africa, 2008–2015

**DOI:** 10.3201/eid2312.162078

**Published:** 2017-12

**Authors:** Marietjie Venter, Marthi Pretorius, James A. Fuller, Elizabeth Botha, Mpho Rakgotho, Voula Stivaktas, Camilla Weyer, Marco Romito, June Williams

**Affiliations:** University of Pretoria Centre for Viral Zoonoses, Pretoria, South Africa (M. Venter, M. Pretorius, E. Botha, M. Rakgotho, V. Stivaktas, J. Williams);; US Centers for Disease Control and Prevention, Global Disease Detection Centre, Pretoria (M. Venter, J.A. Fuller);; National Health Laboratory Service, Tshwane, South Africa (M. Pretorius);; University of Pretoria Equine Research Centre, Pretoria (C. Weyer);; Onderstepoort Veterinary Research, Onderstepoort, South Africa (M. Romito)

**Keywords:** West Nile virus, vector, vector-borne infections, horses, humans, wildlife, neurological disease, South Africa, viruses, livestock, veterinarians, zoonoses

## Abstract

During 2008–2015 in South Africa, we conducted West Nile virus surveillance in 1,407 animals with neurologic disease and identified mostly lineage 2 cases in horses (7.4%, 79/1,069), livestock (1.5%, 2/132), and wildlife (0.5%, 1/206); 35% were fatal. Geographic correlation of horse cases with seropositive veterinarians suggests disease in horses can predict risk in humans.

West Nile virus (WNV) circulates between ornithophilic mosquitoes and birds. Birds are used as WNV sentinels, but in Africa, birds rarely die of infections, probably because of genetic resistance (*1*). Therefore, another animal is needed to predict risk for WNV disease in Africa. Humans and horses are considered incidental dead-end hosts for WNV (*2*). Although ≈20% of infections in these species are symptomatic, ≈1% of human cases involve neurologic disease, with 1%–10% fatality rates (*3*), but 90% of horse cases involve neurologic disease, with 30%–40% fatality rates (*4*). Monitoring equine populations living near human populations might enable prediction of human outbreaks in Africa (*5*).

WNV is endemic to South Africa (*1*). The largest human outbreak occurred in 1974 in Karoo (*1*); during 1983–1984, a smaller epizootic outbreak occurred in Witwatersrand (Gauteng Province) (*6*). During 2010–2011, screening of the cerebrospinal fluid of patients in hospitals in Gauteng Province indicated WNV was present in 3.5% of unsolved cases of neurologic disease, suggesting severe WNV cases might be missed because of a lack of clinical awareness of the pathogenic potential of this virus (*7*).

WNV isolates are divided into 2 lineages (*8*): lineage 1, which predominates in the Northern Hemisphere and Australia, and lineage 2, which is endemic to southern Africa and Madagascar and started emerging in central Europe in 2008 (*9*). Lineage 2‒associated encephalitis outbreaks have been occurring in Greece since 2010, causing hundreds of neurologic cases in humans (fatality rate 17%) and horses (*3*). Lineage 2 was first associated with severe neurologic disease in horses in South Africa in 2009 (*8*). Neurologic signs of infection in horses are similar for both lineages and include ataxia, weakness, recumbence, seizures, and muscle fasciculation (*8*,*10*). However, epidemiologic data are lacking.

South Africa conducts active surveillance for infectious pathogens in horses, such as African horse sickness virus (AHSV), to help the horse industry maintain disease-free status, as required by the World Organisation for Animal Health. Equine WNV is also a World Organisation for Animal Health‒listed disease, enabling researchers to use horses for WNV surveillance internationally. To assist with predicting WNV human cases and managing outbreaks in susceptible animals, we sought to define the epidemiology of WNV in horses in South Africa.

## The Study

We prospectively investigated horses and other animals with fever or neurologic signs during 2008–2015 and compared the geographic range of WNV-positive animals with that of WNV-seropositive veterinarians involved in equine, wildlife, and livestock disease management during 2011‒2012 (*11*). A total of 210 veterinarians from all 9 South Africa provinces submitted blood, neurologic tissue, and visceral tissue specimens from horses (acquired during 2008–2015) and wildlife and livestock (acquired during 2010–2015) that displayed acute fever, neurologic disease, or other signs of acute infection, accompanied by their demographic and disease data, to the Centre for Viral Zoonoses, University of Pretoria (Pretoria, South Africa). A total of 1,407 samples (64% blood/serum, 25% tissue, 6% both blood/serum and tissue, 4% viral RNA) were received.

We tested all specimens for WNV, Shuni virus, alphaviruses, and equine encephalitis virus and submitted samples for rabies virus testing, if suspected, to Onderstepoort Veterinary Research, Onderstepoort, South Africa, as previously described (*12*). All WNV-positive cases were tested for AHSV (*13*). We screened all equine serum and plasma specimens for WNV IgM (WNV IgM Capture ELISA Test; IDEXX Laboratories, Montpellier, France) and confirmed by neutralization assay (*8*). Reverse transcription PCR‒positive cases were sequenced (GenBank accession nos. KY176717‒36) and subjected to maximum likelihood analysis (Technical Appendix Figure). We compared WNV positivity with clinical signs in horses by logistic regression using crude odds ratios (ORs) and adjusted ORs (aORs) with 95% CIs (Stata 14; StataCorp LLC, College Station, Texas, USA) (Technical Appendix Table).

Most clinical cases were in horses (76.0%, 1,069/1,407), followed by wildlife (14.6%, 206/1,407) and livestock (9.4%, 132/1,407). We detected most WNV cases in horses (7.3%, 79/1,069; p<0.001), and 1 (0.5%) case in wildlife (imported North American white-tailed deer [*Odocoileus virginianus*]), and 2 in (1.5%) livestock (locally bred Ayreshire cow [*Bos taurus*], boer goat [*Capra aegagrus hircus*]).

Real-time PCR results were positive for 24 cases; 20 isolates could be sequenced, and 18 clustered with lineage 2 (Technical Appendix Figure). A mare and her miscarried fetus were the only animals infected with lineage 1 viruses (*14*). We detected 14 (17.7%) co-infections in WNV-infected horses (Table 1), with high fatality rates for most co-infecting viruses: MIDV (100%, 4/4); AHSV (66.7%, 2/3); SINV (100%, 3/3); Shuni virus (33.3%, 1/3); and equine encephalitis virus (0%, 0/1).

**Table 1:** WNV infection, co-infection, disease, and death in horses, by year, South Africa, 2008–2015

Category	No. (%) horses								
	2008	2009	2010	2011	2012	2013	2014	2015	Total
Total specimens	71	76	150	164	89	138	193	188	1,069
Confirmed WNV positive†	9 (12.7)	6 (7.9)	18 (12.0)	12 (7.3)	3 (3.4)	4 (2.9)	23 (11.9)	4 (2.1)	79 (7.4)
WNV PCR positive†	5 (7.0)	3 (3.9)	8 (5.3)	2 (1.2)	0 (0)	1 (0.7)	4 (2.1)	1 (0.5)	24 (2.2)
WNV IgM positive†	5 (7.0)	3 (3.9)	12 (8.0)	10 (6.1)	3 (3.4)	3 (2.2)	20 (10.4)	3 (1.6)	59 (5.5)
Deaths‡	5 (55.6)	3 (50.0)	8 (44.4)	3 (25.0)	1 (33.3)	1 (25.0)	5 (21.7)	1 (25.0)	27 (34.2)
Any neurologic signs‡	8 (88.9)	6 (100.0)	16 (88.9)	11 (91.7)	2 (66.7)	4 (100.0)	21 (91.3)	4 (100.0)	72 (91.1)
Fever‡	2 (22.2)	2 (33.3)	3 (16.7)	6 (50.0)	1 (33.3)	1 (25.0)	10 (43.5)	3 (75.0)	28 (35.4)
Co-infections‡ and co-infecting viruses	2 (22.2), 2 AHSV	2 (33.3), 2 SINV	1 (5.6), 1 SHUV	2 (16.7), 2 MIDV	2 (66.7), 1 AHSV, 1 SINV	0	4 (17.4), 2 MIDV, 1 SHUV, 1 EEV	1 (25.0), 1 SHUV	14 (17.7), 3 AHSV, 3 SINV, 3 SHUV, 4 MIDV, 1 EEV

*AHSV, African horse sickness virus; EEV, equine encephalitis virus; MIDV, Middleburg virus; SHUV, Shuni virus; SINV, Sindbis virus; WNV,  West Nile virus. †Percentage of total number of specimens tested. ‡Percentage of total number of confirmed WNV-positive cases. Confirmed cases were those that tested positive by PCR plus those that tested positive by WNV IgM Capture ELISA (IDEXX Laboratories, Montpellier, France) followed by neutralization assay.

**Figure 1:**
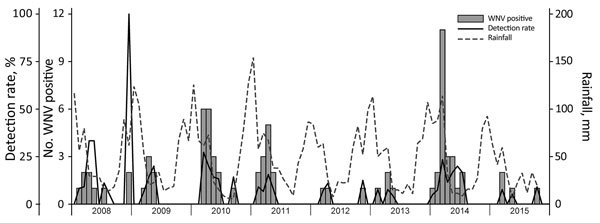
Seasonal occurrence of WNV in horses, South Africa, 2008–2015. Rainfall levels are indicated as a potential correlate for increases in the prevalence of the WNV mosquito vector *Culex univitattus*. WNV, West Nile virus.

**Figure 2:**
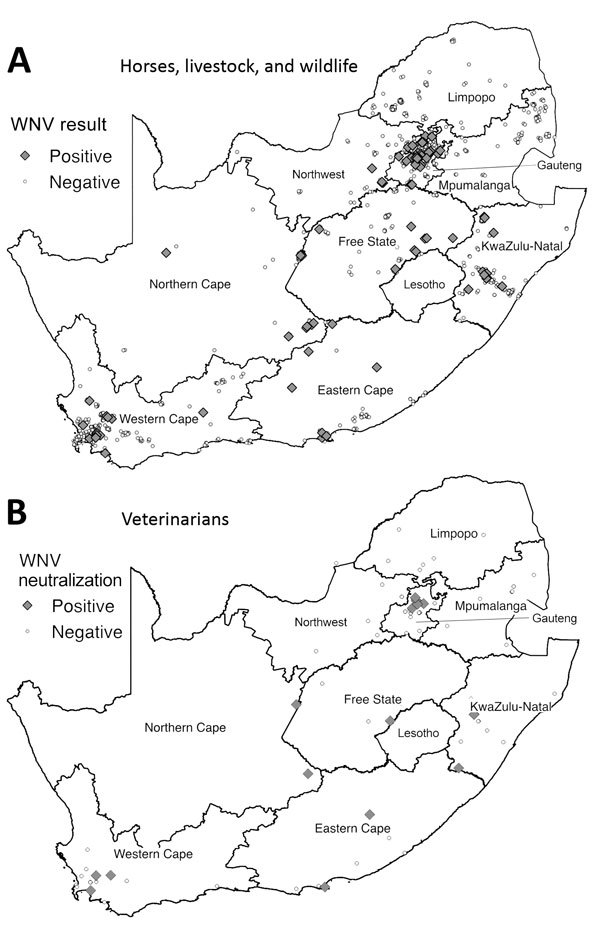
Distribution of WNV cases among horses, livestock animals, and wildlife species during 2008–2015 and of WNV neutralizing antibody‒positive veterinarians involved in equine, wildlife, and livestock disease management during 2011‒2012, South Africa. A) Samples were collected from horses during 2008–2015 and from livestock and wildlife 2010–2015. Samples were considered positive if they tested positive for WNV genome by PCR or for WNV IgM by WNV IgM Capture ELISA (IDEXX Laboratories, Montpellier, France) and WNV neutralizing antibody by neutralization assay. B) Distribution of veterinarians described in previous report (*11*). Human serum was considered positive if virus neutralization was observed at a titer of 1:10 and higher. WNV, West Nile virus.

Most (77.2%) WNV cases occurred in Southern Hemisphere autumn (March‒May) (Figure 1), 2–3 months after peak precipitation. The interannual detection rate among horses was 2.1–12.7% (Table 1). Most specimens came from Gauteng (n = 400) and Western Cape (n = 296) Provinces (Figure 2, panel A). Most WNV-positive cases were from Gauteng Province (7.3%, 29/400), but detection rates were highest in Northern Cape (10.2%) and Eastern Cape (10.5%) Provinces. Of 152 samples from Limpopo and Mpumalanga Provinces, 99 of which came from wildlife species, none were WNV positive. The geographic distribution of WNV-seropositive veterinarians (*11*) was similar to that of WNV-positive horses (Figure 2, panel B).

Older horses (>15 y) were least likely (4.9%, 3/61) and 1–4-year-old horses most likely (41.0%, 25/61) to test WNV positive (Table 2); 35.4% of WNV-positive horses were febrile, 93.7% displayed neurologic signs, and 34.2% died. WNV-associated signs included ataxia, paralysis, paresis, seizures, and tongue paralysis. Multiple logistic regression models (Technical Appendix Table) confirmed neurologic signs as a strong predictor (aOR 4.12, 95% CI 1.59–10.70) and fever a weak predictor (aOR 1.25, 95% CI 0.75–2.06) of WNV positivity. Paresis (aOR 2.74, 95% CI 1.30–5.79) and tongue paralysis (aOR 7.73, 95% CI 1.27–47.18) were both predictive of WNV positivity.

**Table 2:** Characteristics of 1,069 horses positive and negative for WNV infection, South Africa, 2008–2015*

Variable	No./total (%)		Crude OR (95% CI)
	WNV negative, n = 990	WNV positive, n = 79	
Detection method and result			
PCR+ IgM‒	NA	20/79 (25.3)	
PCR‒ IgM+	NA	55/79 (69.6)	
PCR+ IgM+	NA	4/79 (5.1)	
Age, y†			
<1	64/617 (10.4)	6/61 (9.8)	1.00 (Reference)
1–4	170/617 (27.6)	25/61 (41.0)	1.57 (0.62–4.00)
5–9	152/617 (24.6)	15/61 (24.6)	1.05 (0.39–2.84)
10–14	145/617 (23.5)	12/61 (19.7)	0.88 (0.32–2.46)
>15	86/617 (13.9)	3/61 (4.9)‡	0.37 (0.09–1.54)
Died or euthanized§	240/976 (24.6)	27/79 (34.2)	1.59 (0.98–2.59)
Fever¶	389/981 (39.7)	28/79 (35.4)	0.84 (0.52–1.35)
Neurologic signs			
Any neurologic sign#	785/990 (79.3)	74/79 (93.7)	3.86 (1.54–9.68)
Ataxia¶	249/981 (25.4)	21/79 (26.6)	1.06 (0.63–1.79)
Paralysis¶	67/981 (6.8)	10/79 (12.7)	1.98 (0.97–4.01)
Paresis**	71/980 (7.2)	13/79 (16.5)	2.52 (1.33–4.79)
Recumbent¶	105/981 (10.7)	6/79 (7.6)	0.69 (0.29–1.62)
Seizure¶	35/981 (3.6)	4/79 (5.1)	1.44 (0.50–4.16)
Tongue paralysis¶	5/981 (0.5)	2/79 (2.5)	5.07 (0.97–26.56)
Other signs			
Anorexia¶	132/981 (13.5)	6/79 (7.6)	0.53 (0.23–1.24)
Icterus**	63/980 (6.4)	8/79 (10.1)	1.64 (0.76–3.56)
Rectal prolapse¶	6/981 (0.6)	2/79 (2.5)	4.22 (0.84–21.27)

*NA, not applicable; OR, odds ratio; WNV, West Nile virus; +, positive; –, negative. †Data on age were missing for 391 of 1,069 horses. Test for trend p = 0.036. ‡p = 0.035. §Data on this disease characteristic were missing for 14 of 1,069 horses. ¶Data on this neurologic sign were missing for 9 of 1,069 horses. #Defined as the presence of any specific neurologic sign or the clinician indicated that it was a neurologic case without indicating specific neurologic signs. **Data on this neurologic sign were missing for 10 of 1,069 horses.

## Conclusions

This 8-year surveillance confirmed annual WNV outbreaks among horses in South Africa, most cases being lineage 2. The neurologic signs and fatality rate among WNV lineage 2‒infected horses correlated with those described for lineage 1 in Europe and the United States (*4*). Locally bred and imported horses appeared similarly susceptible to WNV neurologic disease. Younger animals were more likely to be infected, although all age groups had fatalities. Generalized neurologic signs, such as paresis and paralysis with death, positively correlated with WNV infection in horses. Fatal WNV encephalitis was diagnosed in a giraffe at Onderstepoort Veterinary Research (M. Romito, unpub. data), suggesting certain wildlife species in Africa might be more susceptible; however, we did not detect WNV-positive local wildlife in our sample. WNV-induced fetal death was recorded only once, with lineage 1 infection, but should continue to be monitored.

The geographic distribution of WNV is mainly dependent on favorable ecology, rainfall, and competent vectors. The range of *Culex univitattus* mosquitoes, the predominant WNV vector, correlated with the geographic distribution of equine cases in South Africa (*6*). The distribution of WNV exposure among horses correlated with that among humans (Figure 2, panels A, B), suggesting horses could serve as sentinels for human risk for WNV disease in South Africa. Horses have low WNV viremia, precluding them from transmitting infections and establishing epidemics in humans; however, those handling horse central nervous system tissue should do so with caution (*15*). Vaccination before the start of the rainy season could reduce the risk for WNV in horses.

In summary, surveillance for neurologic disease in animals across South Africa showed WNV lineage 2 as the primary cause of annual outbreaks, with high fatality rates in horses. Horses proved to be good sentinels for WNV in Africa and can be used to determine geographic and seasonal risk patterns for human WNV disease.

Technical Appendix Association between clinical signs and West Nile virus positivity in horses and maximum likelihood phylogenetic analysis of West Nile virus nonstructural protein 5 gene from isolates acquired in horses.
