# Supplementary material for: West Nile Virus Lineage 2 in Horses and Other Animals with Neurologic Disease, South Africa, 2008–2015
Source: Emerg Infect Dis. 2017 Dec;23(12):2060–4. doi: 10.3201/eid2312.162078 (PMC5708237; doi:10.3201/eid2312.162078)
Supplement: Technical Appendix — Association between clinical signs and West Nile virus positivity in horses and maximum likelihood phylogenetic analysis of West Nile virus nonstructural protein 5 gene from isolates acquired in horses. [file 16-2078-Techapp-s1.pdf]

Article DOI: https://doi.org/10.3201/eid2312.162078

West Nile Virus Lineage 2 in Horses and Other Animals with Neurologic Disease, South Africa, 2008–2015

Technical Appendix

Technical Appendix Table. Association of clinical signs with West Nile virus positivity among 1,069 horses, South Africa, 2008–2015*

	Adjusted odds ratio (95% CI)	
Variable	Model 1	Model 2
Died or euthanized	1.48 (0.90–2.43)	1.42 (0.84–2.39)
Fever	1.25 (0.75–2.06)	0.87 (0.52–1.47)
Neurologic signs		
Any†	4.12 (1.59–10.70)	
Ataxia		0.96 (0.54–1.72)
Paralysis		1.82 (0.78–4.24)
Paresis		2.74 (1.30–5.79)
Recumbent		0.32 (0.11–0.88)
Seizure		1.47 (0.47–4.58)
Tongue paralysis		7.73 (1.27–47.18)
Other signs		
Anorexia		0.40 (0.15–1.04)
Icterus		1.55 (0.63–3.77)
Rectal prolapse		5.93 (0.94–37.26)

*For model 1, death, fever, and the presence of any neurologic sign were used as covariates. For model 2, death, fever, and a specific neurologic and nonneurologic sign were used as covariates.
†Defined as the presence of any specific neurologic sign or the clinician indicated that it was a neurologic case without indicating specific neurologic signs.

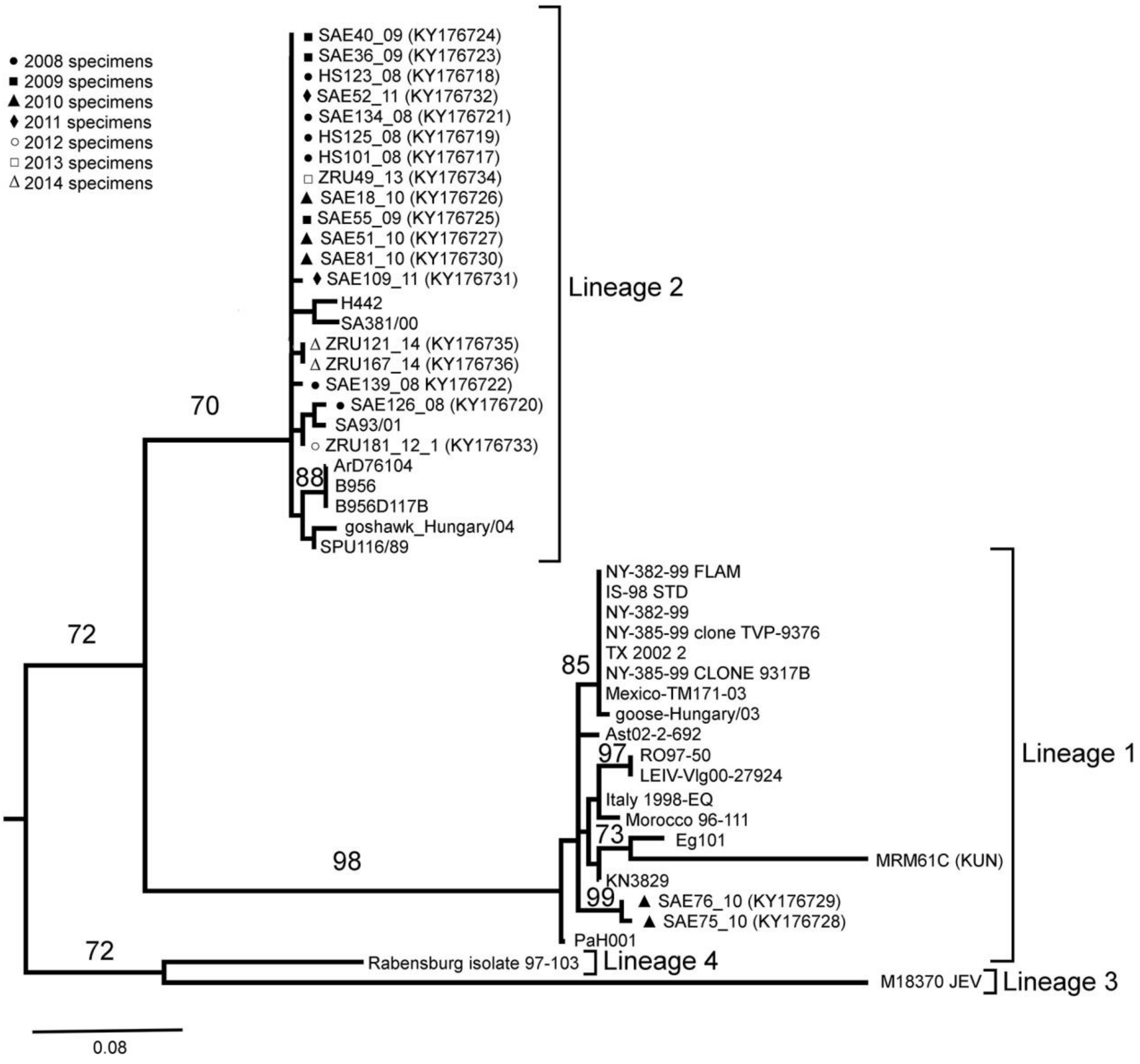

Technical Appendix Figure. Maximum likelihood analysis of West Nile virus (WNV) nonstructural protein 5 (NSP5) region sequences used to determine the lineage of WNV isolates from South Africa, 2008–2015. GenBank accession numbers of study isolates are indicated in parentheses. Maximum likelihood phylogram was recovered from a RAxML analysis excluding invariable sites on the NSP5 region of WNV. Bootstrap support (850 replicates using the autoMRE bootstopping criterion in RAxML) with values $\geq$70 are indicated on branches. JEV, Japanese encephalitis; KUN, Kunjin virus.
